# Human Stool Metabolome Differs upon 24 h Blood Pressure Levels and Blood Pressure Dipping Status: A Prospective Longitudinal Study

**DOI:** 10.3390/metabo11050282

**Published:** 2021-04-29

**Authors:** Justine Huart, Arianna Cirillo, Bernard Taminiau, Julie Descy, Annie Saint-Remy, Georges Daube, Jean-Marie Krzesinski, Pierrette Melin, Pascal de Tullio, François Jouret

**Affiliations:** 1 Division of Nephrology, University of Liège Hospital (ULg CHU), University of Liège, B-4000 Liège, Belgium; A.SaintRemy@chuliege.be (A.S.-R.); JM.Krzesinski@chuliege.be (J.-M.K.); Francois.Jouret@chuliege.be (F.J.); 2 Groupe Interdisciplinaire de Génoprotéomique Appliquée (GIGA), Cardiovascular Sciences, University of Liège, B-4000 Liège, Belgium; 3 Center for Interdisciplinary Research on Medicines (CIRM), Metabolomics Group, University of Liège, B-4000 Liège, Belgium; Arianna.Cirillo@uliege.be (A.C.); P.DeTullio@uliege.be (P.d.T.); 4 Fundamental and Applied Research Center for Animal & Health (FARAH), Veterinary Public Health, University of Liège, B-4000 Liège, Belgium; Bernard.Taminiau@uliege.be (B.T.); Georges.Daube@uliege.be (G.D.); 5 Laboratory for Food Microbiology, Department of Food Sciences, Faculty of Veterinary Medicine, University of Liège, B-4000 Liège, Belgium; 6 Clinical Microbiology, University of Liège Hospital (ULg CHU), University of Liège, B-4000 Liège, Belgium; Julie.Descy@chuliege.be (J.D.); Pierrette.Melin@chuliege.be (P.M.)

**Keywords:** arterial hypertension, dipping status, gut microbiota, short chain fatty acids, acetate, butyrate, propionate, metabolomics, 24 h blood pressure measurement, NMR

## Abstract

Dysbiosis of gut microbiota (GM) has been involved in the pathophysiology of arterial hypertension (HT), via a putative role of short chain fatty acids (SCFAs). Its role in the circadian regulation of blood pressure (BP), also called “the dipping profile”, has been poorly investigated. Sixteen male volunteers and 10 female partners were subjected to 24 h ambulatory BP monitoring and were categorized in normotensive (NT) versus HT, as well as in dippers versus non-dippers. Nuclear magnetic resonance (NMR)-based metabolomics was performed on stool samples. A 5-year comparative follow-up of BP profiles and stool metabolomes was done in men. Significant correlations between stool metabolome and 24 h mean BP levels were found in both male and female cohorts and in the entire cohort (R^2^ = 0.72, R^2^ = 0.79, and R^2^ = 0.45, respectively). Multivariate analysis discriminated dippers versus non-dippers in both male and female cohorts and in the entire cohort (Q^2^ = 0.87, Q^2^ = 0.98, and Q^2^ = 0.68, respectively). Fecal amounts of acetate, propionate, and butyrate were higher in HT versus NT patients (*p* = 0.027; *p* = 0.015 and *p* = 0.015, respectively), as well as in non-dippers versus dippers (*p* = 0.027, *p* = 0.038, and *p* = 0.036, respectively) in the entire cohort. SCFA levels were significantly different in patients changing of dipping status over the 5-year follow-up. In conclusion, stool metabolome changes upon global and circadian BP profiles in both genders.

## 1. Introduction

Modifications of gut microbiota (GM) have been involved in the pathophysiology of various pathologies, such as obesity, diabetes, inflammatory bowel disease, chronic kidney disease, and hypertension (HT), in several preclinical and clinical models [1,2,3,4,5,6,7,8,9,10]. HT is a major cardiovascular risk factor, making it a global public health problem [11]. HT is defined by systolic blood pressure (BP) values ≥140 mmHg and/or diastolic BP values ≥90 mmHg when measured in the office room or ≥130 mmHg and/or ≥80 mmHg when measured with 24 h ambulatory blood pressure monitoring (24 h ABPM). In the vast majority of patients, no cause of HT can be identified, and HT is described as “essential”. The latter results from the combination of multiple genetic and environmental factors of which the GM could be part [1,2,8,11,12]. The absence of a significant drop of night-time BP compared with diurnal BP (which is called “the non-dipping BP profile”) has been associated with poor renal and cardiovascular outcomes [13,14]. The link between GM and BP dipping status is unknown. A better understanding of the pathophysiology of essential HT and the non-dipping BP profile may help identify new therapeutic targets and improve the management of hypertensive patients. The implication of GM-derived metabolites like short chain fatty acids (SCFAs) in BP homeostasis is increasingly recognized [5,15,16,17,18]. SCFAs are the end-products of the bacterial fermentation of carbohydrates contained in food [5]. The respective amounts and ratios between SCFAs depend on the composition of the GM and the diet [19,20,21]. These SCFAs produced in the intestinal lumen are either excreted in faeces or reabsorbed by the intestinal mucosa to end up in the bloodstream, where they participate in host homeostasis by binding G protein-coupled receptors (GPCRs) [5,22,23,24]. SCFAs include mainly acetate, butyrate, and propionate [19]. We and others have suggested a pathophysiological association between GM, fecal SCFAs, and HT in patients [25,26] by demonstrating that HT was significantly associated with higher stool levels of acetate, propionate, and butyrate and that BP levels globally correlated with stool metabolome.

In the present study, we have performed a 5-year longitudinal follow-up of initially non-hypertensive male volunteers (*n* = 16 participants) with a focus on the association between GM, GM metabolome, fecal SCFA levels, and 24 h BP levels (including the BP dipping status) using untargeted nuclear magnetic resonance (NMR)-based metabolomics. Additionally, we have recruited the female partners (*n* = 10 participants) in order to test our hypothesis in both genders and to assess the influence of a similar environment on GM composition and stool metabolome.

## 2. Results

### 2.1. Clinical Characteristics of the Three Cohorts

The flow-chart of patient recruitment is summarized in Figure S1: “2015 male cohort” (*n* = 16); “2020 male cohort” (*n* = 16); and “2020 female cohort” (*n* = 10). Among the 26-participant 2020 male and female cohorts, 6 men and 1 woman were categorized as HT, while the remaining 10 men and 9 women were normotensive (NT). Table 1 summarizes the main clinical features of HT versus NT individuals. No significant difference was found between HT and NT patients concerning the age, BMI, smoking habits, alcohol consumption, family history of HT, diabetes, and number of non-dippers. There was no significant difference between dietary habits in HT versus NT patients (Table S1). As expected, mean 24 h SBP, 24 h DBP, and 24 h MBP levels were statistically higher in HT patients compared with NT controls. Two patients (one man and one woman) were under anti-hypertensive medications: beta-blockers for the man; diuretics and angiotensin convertase enzyme inhibitors for the woman. None of the participants took antibiotics within the 6 weeks preceding the enrolment.

The 2020 male and female cohorts were also categorized into dippers and non-dippers according to 24 h ABPM results. The two patients under anti-hypertensive drugs were excluded from this analysis. Thirteen individuals (*n* = 7 men and 6 women) were categorized as non-dippers (54%). None of our patients had reverse dipping (i.e., a rise of the nocturnal BP). The comparison of the main characteristics between dippers and non-dippers is presented in Table 2. No significant difference was observed concerning the age, BMI, smoking habits, alcohol consumption, family history of HT, and diabetes. As expected, the night–day SBP ratios were significantly different between dippers and non-dippers. There was no significant difference between dietary habits in dippers versus non-dippers (Table S2). In addition, there was also no significant difference in the main clinical characteristics (including BP levels) between dippers and non-dippers when studying only NT patients (data not shown). As the HT group included too few patients, the analysis was not done.

Table 3 compares the clinical characteristics between “2015 male cohort” and “2020 male cohort”, with emphasis on the six patients who changed their BP status over time. The mean time between the first 24 h ABPM and the second one was 4.8 years (standard deviation = 4 months). There was no significant difference (in terms of BMI, smoking habits, alcohol consumption, and non-dipping BP profile) between 2015 and 2020 for the 16 patients. Note that the male volunteer under beta-blockers for cardiac palpitations was already treated in 2015. Table S3 compares the 24 h ABPM results between “2015 male cohort” and “2020 male cohort” for each patient. Six patients changed BP status: 2 HT became NT and 4 NT became HT. Six patients changed BP dipping status: 2 non-dippers became dippers and 4 dippers became non-dippers. Concerning the dietary habits among the six patients who changed their BP status, one NT patient became vegetarian and HT in 2020 and one HT patient decreased his salt consumption and became NT in 2020. Concerning the entire male cohort, one patient ceased to be vegetarian and his BP status remained stable.

Table S4 compares the clinical characteristics between the “2020 male cohort” and the “2020 female cohort”. BMI, alcohol consumption, and mean 24 h DBP were significantly higher in the male cohort. There were, however, no significant differences in terms of smoking habits, family history of HT, gastroenterological and cardiovascular diseases, diabetes, mean 24 h SBP, mean 24 h MBP, and non-dipping BP profile. When comparing the dietary habits of the two members of the same couple, one couple had significantly different habits: one was vegetarian and the other not. The other nine couples did not have any significant difference in their dietary habits.

### 2.2. Anaerobic Culture of Fecal Bacteria

The main anaerobic bacteria present in the stools of the 2020 male and female cohorts were the Bacteroidetes vulgatus and uniformis, the Bifidobacterium longum, and the Collinsella aerofaciens. There was no significant difference for the presence of these bacteria in faeces between HT versus NT patients, as well as between dippers versus non-dippers.

### 2.3. GM Characterization by 16S Amplicon Sequencing

The 16S amplicon sequencing included 10,000 identifications by sample, reaching a mean Good’s coverage value of 99.62% at the genus level. We focused on phylum, family, genus, and species. Only the bacterial populations with a median value different from zero among the entire cohort were considered. The most abundant phyla in the global cohort included Firmicutes (59.26%), Bacteroidetes (32.47%), Proteobacteria (2.94%), and Verrucomicrobia (2.33%). Concerning families, microbiota profiles were dominated by four families, Lachnospiraceae (mean relative abundance: 26.29%), Ruminococcaceae (22.29%), Prevotellaceae (16.59%), and Bacteroidaceae (12.46%). Paired analyses showed that GM composition remained stable from 2015 to 2020 in the male cohorts. No significant change was observed in patients who changed their BP or dipping status between 2015 and 2020. ANOVA followed by Tukey post-hoc test failed to show statistically significant differences between bacterial phyla, families, genera, and species among HT versus NT patients or among dippers versus non-dippers in the 2020 entire cohort. No significant correlation between genera or species and the 24 h MBP levels was found. There was no significant difference in the GM of men versus women in the 2020 cohorts. Regarding the dissimilarity of the GM, there was no significant difference between couples who shared the same BP (or dipping status) or not. The cumulated relative population abundance for the genera contributing to more than 1% of the GM composition is reported in Figure S2A for all patients from 2020 cohorts. Figure S2B shows the comparison between the GM profile of male patients in 2015 versus 2020.

### 2.4. Untargeted Metabolomics: Multivariate Analyses of the Entire Stool Metabolome

**Figure 1:**
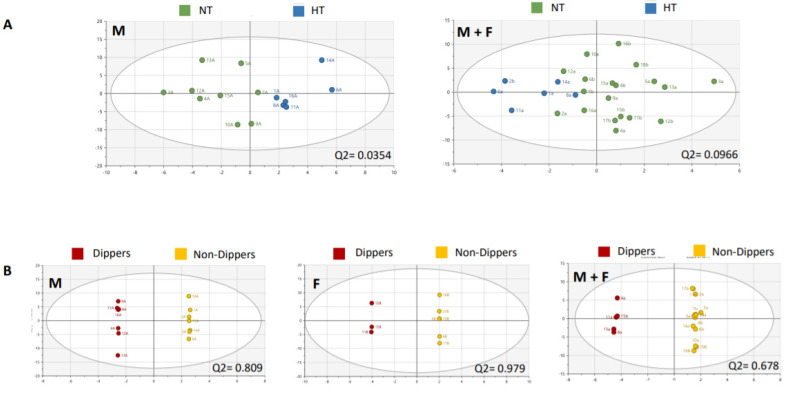
Discriminant analyses of stool metabolomes. (**A**) Discriminant analysis (principal component analysis (PCA)-X) between normotensive (NT) and hypertensive (HT) groups in the 2020 male cohort (M) and in the entire cohort (M + F) (number of components = 2; number of components = 4). (**B**) Discriminant analysis (orthogonal partial least squares discriminant analysis (OPLS-DA)) between dippers and non-dippers in the 2020 male cohort (M), the 2020 female cohort (F) and in the entire cohort (M + F). Significance level was set for Q^2^ ≥ 0.5.

**Figure 2:**
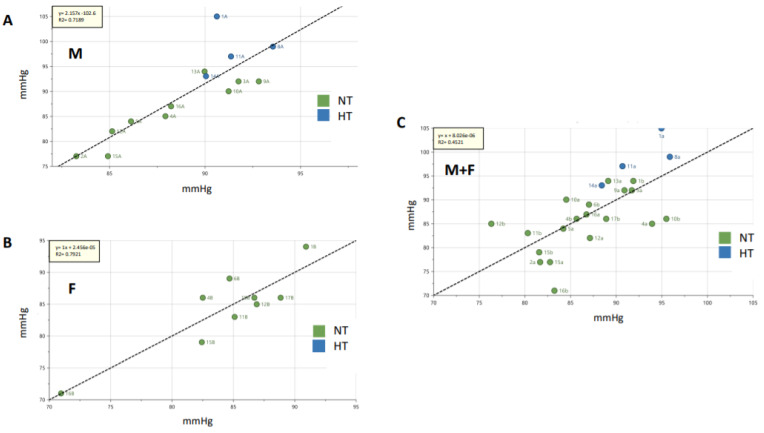
Partial least square regression lines for stool metabolomes. The regression analysis highlighted a good linear correlation with 24 h MBP levels: (**A**) in the 2020 male (M) cohort (R^2^ = 0.72) and (**B**) in the 2020 female (F) cohort (R^2^ = 0.79). The correlation was less significant in the entire cohort (M + F) (R^2^ = 0.45, (**C**)). The X axis represents the predicted 24 h MBP values based on the fecal metabolomes and the Y axis represents the actual 24 h MBP values. The units for both axes are expressed in mmHg. The significance level was set for R^2^ ≥ 0.4.

An untargeted metabolomics approach was applied to the stool samples using NMR. Multivariate analysis of stool metabolomes did not discriminate male from female groups in the 2020 cohort, even after using supervised methods (Q^2^ at −0.0364; *n* = 18 (Figure S3)). Multivariate analysis (principal component analysis (PCA)-X and orthogonal partial least squares discriminant analysis (OPLS-DA)) of stool metabolomes data were not discriminant between HT versus NT individuals in the 2020 male cohort (Q^2^ at 0.0354; *n* = 15), as well as in the entire cohort (Q^2^ = at 0.0966; *n* = 25) (Figure 1A). However, multivariate analysis (OPLS-DA) of stool metabolomes data was discriminant between dippers versus non-dippers in both 2020 male and female cohorts (Q^2^ = 0.809; *n* = 14 and 0.979; *n* = 9, respectively) and in the entire 2020 cohort (Q^2^ = 0.678; *n* = 18) (Figure 1B). For both OPLS-DA discriminant analyses of HT versus NT individuals and dippers versus non-dippers, a list of VIP was generated (Table 4). In this listing, acetate, propionate, and butyrate were identified and reported several times (different ppm corresponding to signals of the same metabolite), in contrast to the other features that were not identified as belonging to the matrix. After identification of the relevant features by means of correlation spectroscopy (COSY), heteronuclear single quantum coherence spectroscopy (HSQC) and HMDB, Chenomx, integration of acetate, propionate, and butyrate signals was done using maleic acid as internal standard. As previously described, significant correlations between stool metabolomes and 24 h MBP levels were found in 2020 male and female cohorts (R^2^ = 0.7189; *n* = 14 and R^2^ = 0.7921; *n* = 9, respectively). However, less significant correlation was found after considering the entire 2020 cohort (R^2^ = 0.4521; *n* = 23) (Figure 2). The dietary survey showed fairly similar diets between members of the same couple, except for one couple, who were excluded from this analysis. By considering each couple as a group, the variation between the individuals belonging to the same cluster, named “inertia within group”, was measured. In this case, a higher inertia within the group was found in stool metabolomes of couples with a different BP status (51.4% of inertia; *n* = 8) compared with the couples in whom both individuals presented the same BP status (37.5% of inertia; *n* = 10). No difference was observed in the inertia concerning the dipping status within one given couple. Concerning the longitudinal approach, PCA shows a clear separation (R^2^ = 0.709) between the whole stool metabolomes data of 2015 and 2020, which hampered any merge and comparative analyses (data not shown).

### 2.5. Metabolomics: Univariate Analyses of the Relative Quantification of the Three Main SCFA Levels

**Figure 3:**
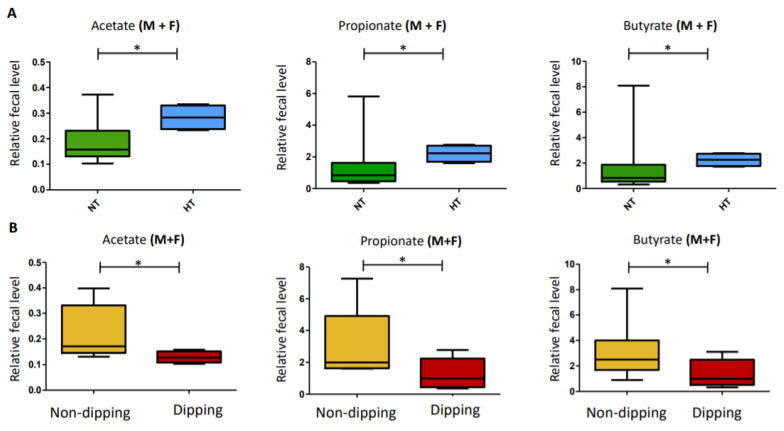
Relative quantification of the three main short chain fatty acids (SCFAs). (**A**) Relative amounts of acetate, propionate, and butyrate in the stools of HT versus NT patients in the entire cohort (Male (M) + Female (F)). (**B**) Relative amounts of acetate, propionate, and butyrate in the stools of dippers versus non-dippers in the entire cohort (Male (M) + Female (F)). The significance level was set at the 5% level. * 0.01 > *p* < 0.05.

**Figure 4:**
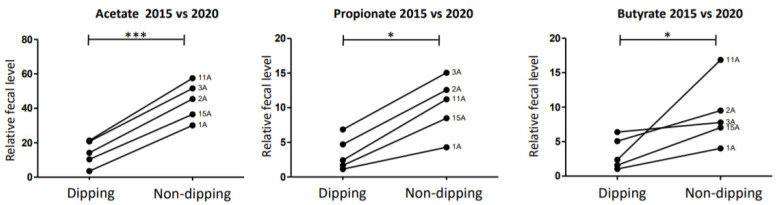
Trajectory of SCFAs according to dipping status between 2015 and 2020. Significant changes were noticed in the acetate, propionate, and butyrate levels in patients who changed their dipping status between the two periods (*p* ≤ 0.0001, *p* = 0.0317, and *p* = 0.0317; *n* = 10), with higher levels of SCFAs when the patients were non-dippers compared with when they were dippers. The significance level was set at the 5% level. *** *p* < 0.001; * 0.01 > *p* < 0.05.

Fecal amounts of acetate, propionate, and butyrate were higher in HT patients than in NT patients in the entire cohort (*p* = 0.0475, *p* = 0.038, and *p* = 0.038, respectively) (Figure 3A). Fecal amounts of acetate, propionate, and butyrate were also significantly higher in non-dippers versus dippers in the entire cohort (*p* = 0.033, *p* = 0.038, and *p* = 0.036, respectively) (Figure 3B). From 2015 to 2020, six patients changed their BP status. However, no significant change was noted in the acetate, propionate, or butyrate levels in these patients between the two periods (*p* = 0.71; *p* = 0.55 and *p* = 0.84, respectively; *n* = 10) (Figure S4). From 2015 to 2020, six patients changed their dipping status as well. Significant changes were noted in the acetate, propionate, and butyrate levels between the two periods (*p* ≤ 0.0001, *p* = 0.032, and *p* = 0.032; *n* = 10), with higher levels of these SCFAs when the patients were non-dippers compared with when they were classified as dippers (Figure 4).

## 3. Discussion

We have recently reported on significant correlations between 24 h MBP levels and the Clostridial order [25]. These correlations were consistent with the CARDIA study involving 529 patients (53.9% of women and mean age of 55.3 years), which highlighted a similar positive correlation between Clostridium IV genus and HT [26]. In the present follow-up study, we did not find significant correlations between BP levels and bacterial genus or species. We did not find significant differences between either HT and NT patients or between dippers and non-dippers concerning the 16S amplicon sequencing results or the anaerobic culture. This is most probably due to the limited number of patients. After a mean follow-up period of 4.8 years, we did not detect significant changes in either the composition of GM of the 16 male volunteers, despite modifications of BP, or the dipping status in some of them. This observation supports the relative stability of GM composition over time in adults [27]. However, such a stability may also be due to the small size of our cohort and/or to too slight BP changes with no significant impact on GM composition.

By contrast, we confirm correlations between 24 h MBP and the entire stool metabolome. These correlations were observed in both genders. Note that correlations were stronger in female and male groups taken separately compared with the entire group, which may suggest differences in metabolome composition between male and female patients. The increased inertia within couples with a different BP status strengthens the putative link between BP homeostasis and the stool metabolome, as this analysis indirectly attenuates the weight of the environmental external factors, like the diet of the daily way of living. This result should nevertheless be tempered given the small number of couples included in the present analysis.

In stools of HT patients, higher levels of SCFAs have been previously measured by gas or liquid chromatography/mass spectrometry [17,28] as well as by 1H-NMR [25]. Similar findings have been reported in stools of rats affected by HT caused by high-salt diet [29]. Such an intestinal accumulation of SCFAs may be secondary to a lower SCFA absorption by the intestinal mucosa via a direct and/or indirect effect of dysbiosis on gut wall permeability [29]. In the present 26-patient cohort, an increased abundance of SCFAs was noted in the stools of HT patients compared with NT controls in both genders. Unfortunately, we could not merge and compare the stool metabolomes of the 2015 versus 2020 collections. This could be due to sampling, pre-analytical, and/or analytical differences, as well as real changes in GM metabolome composition. This highlights the challenges and difficulties in using GM metabolites as follow-up markers for BP. However, the acetate, propionate, and butyrate fecal abundances seem to change over time in patients who changed their dipping status between 2015 and 2020, with higher levels associated with of these bacteria the non-dipping BP profile. A similar evolution in SCFA levels was not observed in patients who changed their BP status between 2015 and 2020, although the correlation between 24 h MBP levels and the entire stool metabolome remained significant despite the variations in BP levels.

Finally, a strong association between the non-dipping BP profile and the stool metabolome, including the relative fecal amounts of acetate, propionate, and butyrate, was observed in both genders. To the best of our knowledge, the BP dipping status has never been associated thus far with stool metabolome. One recent study performed on Dahl salt-sensitive rats has highlighted some correlations between fecal bacterial taxon (e.g., Sutterella) and the BP dipping of the animals [30]. Our group has recently reported on a cohort of 44 patients in which higher fecal SCFAs amounts were found in non-dippers compared with dippers [31]. The non-dipping BP pattern reflects a disruption in the circadian BP rhythm [32,33]. The metabolic abnormalities leading to non-dipping BP profile have been recently linked to altered molecular components of the circadian timing system controlled by the central clock in the hypothalamus and by peripheral clocks in the heart, kidneys, and vessels [32]. The circadian misalignment between peripheral and central clocks is not well understood, but it may implicate changes in GM [34,35]. Indeed, 60% of the total gut bacteria undergo circadian oscillations in their relative abundance mostly influenced by the host daily feeding/fasting cycle, which may be disrupted in the case of circadian misalignment and/or dysbiosis. These circadian oscillations in GM composition may also lead to rhythmic secretion of metabolites [34]. Inversely, integrity of GM and its secretion of metabolites (including SCFAs) seem to be essential for physiological circadian rhythmicity of gene expression in the intestinal epithelium, as well as in hepatocytes [36,37]. Thaiss et al. showed in both mice and humans that jetlag-induced dysbiosis promotes the development of obesity and glucose intolerance, which are transferrable through fecal transplantation to germ-free mice [38]. On the basis of these observations, one may speculate that disruption in the circadian BP rhythm (i.e., non-dipping BP) may be associated with a disrupted rhythmicity of GM-mediated metabolite secretion, including SCFAs. None of our patients presented metabolic dysfunctions or confounding factors when comparing dippers versus non-dippers. Note that assessing the dipping profile of a patient shows an imperfect reproducibility of ~80% and highly depends on the quality of the sleep [39,40,41]. The quality of sleep in our cohort at the time of BP monitoring was reported as moderately good. Although confirmatory data in larger cohorts are obviously needed with repeated dipping status assessments, our observation of a putative link between GM and stool metabolome and BP dipping may unravel innovative pathophysiological investigations in the field.

In conclusion, our longitudinal 26-patient cohort with a mean follow-up of ~5 years confirms that the fecal metabolome is associated with 24 h MBP levels in both genders, with higher SCFA levels in the faeces of HT patients. There was no significant change in GM composition. The “function” of GM (i.e., the secretion of metabolites) seems then to be more modified over time in patients changing their BP levels than the composition of the GM itself. This hypothesis has to be confirmed using metatranscriptomics and/or metaproteomics approach to better assess metabolites production. Our data highlight a novel putative link between the stool metabolome (and specifically SCFAs) and the non-dipping BP profile. Moreover, our longitudinal study demonstrated that a modification of fecal SCFA levels is associated with a modulation of the dipping status of patients. Further investigations, including interventional trials aiming at evaluating the hypotensive effect of GM modifications and/or modulation of the fecal metabolome by pre-biotics, pro-biotics, or post-biotics (such as SCFAs per se), are needed to confirm our exploratory data. One clinical trial (registered under the following number: NCT04415333) plans to evaluate the effect that butyrate absorbed in the gut (via the participant self-administering an enema with butyrate) has on BP in African Americans. This study is not yet recruiting and should start in May 2021.

## 4. Materials and Methods

The present single-center prospective study was approved by the institutional review board of the University of Liège on 20 May 2019 (ethical code number: B707201318600). All new raw sequencing libraries have been deposited at the National Center for Biotechnology Information (NCBI) and are available under the Bioproject PRJNA683149. Stool based Amplicon libraries obtained from the 2015 male cohort can be found under Bioproject PRJNA507937. Metabolomics data have been uploaded to Metabolights depository [42].

*Patients.* The non-hypertensive male volunteers of our princeps (i.e., 2015 male cohort, *n* = 16) and their female partners (*n* = 10) were contacted to form the “2020 male cohort” and “2020 female cohort”, respectively [25]. After signed informed consent, 24 h ABPM (Spacelabs 90,207 device) was performed. BP was measured every 20 min during the day and every 30 min during the night. Mean day-time and night-time systolic BP (SBP), diastolic BP (DBP), and mean BP (MBP) levels were calculated on the basis of self-declared awake and asleep periods. MBP was calculated by Spacelabs at each BP measurement according to the following formula: MBP = DBP + 1/3(SBP-DBP). Participants were divided into two groups: normotensive (NT) or HT, based on European Society of Hypertension (ESH) criteria [43]. NT was defined by mean 24 h BP levels <130/80 mmHg in untreated individuals with or without isolated nocturnal HT (≥120 mmHg (systolic) and/or ≥70 mmHg (diastolic)), but without isolated daytime HT (≥135 mmHg (systolic) and/or ≥85 mmHg (diastolic)). HT was defined by mean 24 h BP levels ≥130 mmHg (systolic) and/or ≥80 mmHg (diastolic) or by isolated daytime HT (≥135 mmHg (systolic) and/or ≥85 mmHg (diastolic)) or in the case of use of antihypertensive medications, regardless of the BP levels. In our previous study, 9 patients among the 16 non-hypertensive ones were considered as “borderline” (mean 24 h BP levels <130/80 mmHg with either isolated night-time or daytime HT). For this study, they were recategorized as NT or HT according to the above criteria. A patient was categorized as dipper when his night–day SBP ratio was ≤0.9 or non-dipper when his night–day SBP ratio was >0.9 [44]. The quality of sleep during the monitoring was evaluated by a questionnaire. All patients had to complete a form including body mass index (BMI), medical history, treatment, addictions, and dietary habits. The following questionnaire about the diet was used:

- Do you eat white or whole wheat bread, rice, and pasta? (White/Whole wheat/Both)
- Do you eat yogurt? (Yes/Never)
- Do you follow a vegetarian diet? (Yes/No)
- Do you eat fruits and vegetables daily? (Yes/No)
- What kind of fats do you eat? (Butter/Vegetable oil/Both)
- Do you consume sugar or sweeteners? (Sugar/Sweetener/Both)
- Do you use salt for cooking? (Yes/Never).

Dietary habits were considered significantly different between two individuals of the same couple if at least three items from the questionnaire obtained a different response or if one of the two individuals was vegetarian and the other not.

*Samples.* Feces were collected at home using stool collection tubes provided with the PSP Spin Stool DNA Plus Kit (ISOGEN Life Science) for GM composition analysis and Fecal Swab Collection^®^ tubes for the anaerobic cultures and the metabolomics study. The collectors for stool sampling were given at the time of ABPM onset (day 1) and were brought back the next day (day 2, when ABPM device was removed). Samples in stool collection tubes were immerged with stool DNA stabilizer solution [45] and stored at −80 °C. Samples in Fecal Swab Collection^®^ tube were cultured in anaerobic conditions following a 10 s vortex and withdrawal of the swab from the cap and the rest of the stools were centrifuged for 10 min at 3000 rpm. Supernatants were aliquoted and stored at −80 °C for the metabolomics analysis.

*Anaerobic culture of stool samples*. Semi-quantitative inoculation of stool samples was performed by streaking. We used three different agars: SCH (Schaedler medium, non-selective type for anaerobic bacteria), KV (medium supplemented with kanamycin and vancomycin, for the selective isolation of Gram-negative anaerobic bacteria), and CNA (medium supplemented with colimycin and nalidixic acid for the selective isolation of Gram-positive bacteria). Incubation in anaerobic jar for 48 h at 37 °C was done before reading the cultures. Species identification of the predominant colony type was performed using MALDI-TOF (Matrix Assisted Laser Desorption Ionization Time-Of-Flight) mass spectrometry.

*16S Amplicon sequencing.* Total bacterial DNA was extracted using the PSP Spin Stool DNA Plus Kit following the manufacturer’s recommendations. PCR-amplification of the V1-V3 region of the 16S ribosomal DNA (rDNA) and library preparation were performed with the following primers (with Illumina overhand adapters): forward (5′-GAGAGTTTGATYMTGGCTCAG-3′) and reverse (5′-ACCGCGGCTGCTGGCAC-3′). Each PCR product was purified with the Agencourt AMPure XP beads kit (Beckman Coulter, Pasadena, CA, USA) and submitted to a second PCR round for indexing, using the Nextera XT index primers 1 and 2. After purification, PCR products were quantified using the Quant-IT PicoGreen (ThermoFisher Scientific, Waltham, MA, USA) and diluted to 10 ng/µL. A final qPCR quantification of each sample in the library was performed using the KAPA SYBR^®^ FAST qPCR Kit (KapaBiosystems, Wilmington, MA, USA) before normalization, pooling and sequencing on a MiSeq sequencer using v3 reagents (ILLUMINA, Illumina Netherlands, Eindhoven, The Nederlands). Positive control using DNA from 20 defined bacterial species and a negative control (from the PCR step) were included in the sequencing run.

*Microbiota profiling.* Sequence read processing was used as previously described [46] using MOTHUR software package v1.40 [47] and VSEARCH algorithm [48,49] for alignment, operational taxonomic unit (OTU) clustering, and chimera detection, respectively. A clustering distance of 0.03 was used for OTU generation. 16S rDNA reference alignment and taxonomical assignation were based upon the SILVA database (v1.32) of full-length 16S rDNA sequences [50]. Subsample datasets of 10,000 reads per sample were obtained using MOTHUR and used to evaluate Good’s sampling coverage and ecological indicators: richness estimation (Chao1 estimator), microbial biodiversity (reciprocal Si mpson index), and the population evenness (derived from Simpson index), using MOTHUR. Population structure and community membership were assessed with MOTHUR using dissimilarity matrix based on the Bray–Curtis dissimilarity index (a measure of community structure that considers shared OTUs and their relative abundances) [51]. Ordination analysis and 3D plots were performed with Vegan, Vegan3d, and rgl packages in R. Non-metric dimensional scaling based upon the Bray–Curtis dissimilarity matrix was applied to visualize the biodiversity between the groups using MOTHUR [52].

*1H-Nuclear Magnetic Resonance data acquisition.* Here, 600 µL of stool supernatants was centrifuged for 6 min at 13,300 rpm (4 °C) to eliminate membranes and cell residues. Then, 400 µL of supernatant was supplemented with 200 μL of deuterated phosphate buffer (DPB, pH 7.4), 100 μL of a 5 mM solution of maleic acid, and 10 μL of a 10 mg/mL TMSP D2O solution. All samples were recorded at 298 K on a Bruker Neo spectrometer operating at 500.13 MHz for the proton signal acquisition. The instrument was equipped with a 5 mm TCI cryoprobe with a Z-gradient. Maleic acid was used as internal standard for quantification and trimethylsilyl-3-propionic acid-d4 (TMSP) for the zero calibration. 1H-NMR spectra were acquired using CPMG relaxation-editing sequence with presaturation for stool supernatants. The CPMG experiment used a RD-90-(t-180-t)n-sequence with a relaxation delay (RD) of 2 s, a spin echo delay (t) of 400 ms, and the number of loops (n) equal to 80. The water suppression pulse was placed during the relaxation delay (RD). The acquisition time was set to 3.982555 s. For all samples, the number of transients was typically 64 and a quantity of four dummy scans was chosen. The data were processed using TopSpin software (version 4.0.8; Bruker Biospin, Rheinstetten, Germany). Phase and baseline corrections were performed manually over the entire range of the spectra and the δ scale was calibrated to 0 ppm using the internal standard TMSP.

*Untargeted metabolomics.* Before multivariate analysis, the spectral intensities of the optimized 1H-NMR spectra were normalized to total intensities and reduced to integrated regions of equal width (0.02 ppm) corresponding to the 0.5–10.00 ppm region. Because of the residual signals of water and maleic acid, regions between 4.7 and 5 ppm (water signal) and 5.6–6.2 ppm (maleic acid signal) were removed before analysis using MestReNova software (v14.1.1). Once the spectra were processed, multivariate analysis (principal component analysis (PCA)-X and orthogonal partial least squares discriminant analysis (OPLS-DA)) models of stool metabolomes were used to identify outliers and separation between the groups, respectively. From the OPLS-DA discriminant model, a list of variable importance of projection (VIP) was generated. VIP-scores larger than 1 indicate “important” X-variables for the discrimination in classes. For evaluating if the diet influences the stool metabolome more than the BP status or the non-dipping BP profile, data analyses were performed within the couples by separating the samples into two groups: couples in which male and female have the same BP or dipping status and couples in which male and female have a different BP or dipping status, respectively. Couples who had significantly different dietary habits were excluded from these analyses. The measure of inertia within a group was used to represent the variations between the stool metabolomes of individuals belonging to the same couple. For univariate analysis of SCFAs, spectral data of stool samples were used to provide a relative quantification of three pre-selected SCFAs: acetate, butyrate, and propionate concentrations were obtained by the integration of the signals at 1.93 ppm, 1.56 ppm, and 1.05 ppm, respectively, using maleic acid as internal standard and Topspin software (version 4.0.8; Bruker). As acetate was also present in small quantities in the conservation media of Fecal Swab Collection^®^ tube, its signal was normalized according to the peak at 8.46 ppm, a metabolite exclusively present in the matrix.

### Statistical Analysis

*Patients.* Continuous variables were expressed as mean ± standard deviation; frequencies of categorical variables were expressed as percentages. Mann–Whitney U test and Yates Chi-square test were used to compare continuous variables and categorical variables, respectively, between two groups. Comparisons of paired groups were performed using Wilcoxon and McNemar tests with continuity correction for continuous variables and categorical variables, respectively. Significance was set at the 5% level. Tests were performed with Statistica software (v 13). Patients under antihypertensive medications were excluded from the analyses concerning the BP dipping status and the 24 h MBP levels.

*Microbiology.* Fisher’s exact test was used to compare the presence of the predominant fecal anaerobic bacteria between two groups.

*16S amplicon sequencing.* Statistics for bacterial biodiversity, richness, and evenness were assessed with two-way analysis of variance (ANOVA) corrected for multi-testing (Benjamini, Krieger, and Yekutieli). Statistical differences between groups of specific bacterial populations were assessed by two-way ANOVA and Tukey–Kramer post-hoc test. Unpaired t-test was used to make comparisons between two groups. Tests were performed using PRISM 8 (Graphpad Software).

*Metabolomics*. For univariate analysis of SCFAs, a Mann–Whitney test was used for comparisons between two groups. Comparisons of paired groups were performed using Wilcoxon test. For multivariate analysis, the reduced and normalized NMR spectral data were imported into SIMCA (version 13.0.3, Umetrics AB, Umea, Sweden). Pareto scaling was applied to bucket tables and discriminant analyses (DA), such as PCA, PLS-DA (partial least squares discriminant analysis), OPLS-DA, and PLS (partial least square) regression, were performed. SIMCA was used to generate all PCA, PLS, and PLS-DA models and plots. PCA was only used to detect possible outliers and determine intrinsic clusters within the data set, while PLS-DA maximized the separation. Metabolomics data were subjected to a Tukey test for multiple comparisons. Of technical note, visual aberrant spectral data were considered as outliers and removed from the analyses.

## Figures and Tables

**Table 1:** Clinical characteristics of the 2020 male and female cohorts (normotension vs. hypertension).

2020 Male and Female Cohorts	Normotension	Hypertension	*p* Value
**N**	19	7	
Age (years)	49.2 ± 13.7	52.5 ± 10.5	0.469
Female (N)	9	1	
BMI (kg/m^2^)	23.3 ± 2.5	25 ± 2.9	0.132
Smokers (%, N)	5% (1)	14.3% (1)	0.949
Alcohol (glass/week)	3.7 ± 6	6 ± 7	0.193
Family HT (%, N)	37% (7)	57% (4)	0.629
Diabetes (%, N)	0	14.3% (1)	0.595
CV history (%, N)	16% (3)	29% (2)	0.862
GE history (%, N)	36.8 (7)	14.3 (1)	0.531
24 h Systolic BP (mmHg)	113 ± 7	127 ± 4	0.0005
24 h Diastolic BP (mmHg)	70 ± 6	81 ± 4	0.0004
24 h Mean BP (mmHg)	82 ± 6	98 ± 5	0.0005
Anti-HT treatment (%, N)	0	28.6% (2)	ns
Non-dippers (%, N)	58% (11)	57% (4)	0.679

BMI: body mass index; BP: blood pressure; CV: cardiovascular; GE: gastroenterological; HT: hypertension; ns: not significant. Continuous variables are expressed as mean ± standard deviation. Frequencies of categorical variables are expressed as percentages and the exact number of patients is indicated in brackets. Mann–Whitney U test and Yates Chi-square test were used to compare continuous variables and categorical variables, respectively. Significance was set at the 5% level.

**Table 2:** Clinical characteristics of the 2020 male and female cohorts (dippers vs. non-dippers).

2020 Male and Female Cohorts	Dippers	Non-Dippers	*p* Value
N	11	13	
Age (years)	48.8 ± 11.4	51.9 ± 14.6	0.643
Female (N)	3	6	
BMI (kg/m^2^)	23.6 ± 2.5	23.5 ± 3	0.907
Smokers (%, N)	9% (1)	0	ns
Alcohol (glass/week)	6 ± 8	3.4 ± 4.8	0.417
Family HT (%, N)	63.6% (7)	23% (3)	0.111
Diabetes (%, N)	0	0	ns
CV history (%, N)	0	23% (3)	0.278
GE history (%, N)	36.4% (4)	30.8% (4)	0.884
24 h Systolic BP (mmHg)	119 ± 9	114 ± 9	0.131
24 h Diastolic BP (mmHg)	74 ± 7	71 ± 8	0.417
24 h Mean BP (mmHg)	90 ± 8	87 ± 9	0.602
ND Systolic BP ratio	0.85 ± 0.03	0.93 ± 0.03	<0.0001

BMI: body mass index; BP: blood pressure; CV: cardiovascular; GE: gastroenterological; HT: hypertension; ND: night–day; ns: not significant. Continuous variables are expressed as mean ± standard deviation. Frequencies of categorical variables are expressed as percentages and the exact number of patients is indicated in brackets. Mann–Whitney U test and Yates Chi-square test were used to compare continuous variables and categorical variables, respectively. Significance was set at the 5% level.

**Table 3:** Comparison of the clinical characteristics between the “2015 male cohort” and the “2020 male cohort”.

All Patients	2015 Male Cohort	2020 Male Cohort	*p* Value
N	16	16	
Age (years)	47.6 ± 12.2	52.3 ± 12.4	0.0004
BMI (kg/m^2^)	23.8 ± 2.4	24.6 ± 2.6	0.214
Smokers (%, N)	12.5 (2)	6.2 (1)	ns
Alcohol (glass/week)	4.8 ± 3.5	6.4 ± 7	0.272
Family HT (%, N)	50 (8)	50 (8)	0.479
Diabetes (%, N)	0	6.2 (1)	ns
CV history (%, N)	12.5 (2)	18.7 (3)	ns
GE history (%, N)	31.2 (5)	31.2 (5)	ns
Anti-HT treatment (%, N)	6.2 (1)	6.2 (1)	ns
Non-dippers (%, N)	31.2 (5)	50 (8)	0.449
**Patients with Change in BP Status**	**2015 Male Cohort**	**2020 Male Cohort**	***p* Value**
**N**	6	6	
Age (years)	42.6 ± 13.6	47.1 ± 13.2	0.027
BMI (kg/m^2^)	22.6 ± 1.7	23.5 ± 1.7	0.345
Smokers (% N)	33 (2)	16.6 (1)	ns
Alcohol (glass/week)	4.7 ± 1.4	7.3 ± 7	0.345
Family HT (%, N)	66 (4)	66 (4)	ns
Diabetes (%)	0	0	ns
CV history (%)	0	0	ns
GE history (%, N)	16.6 (1)	16.6 (1)	ns
Anti-HT treatment (%)	0	0	ns
Non-dippers (%, N)	50 (3)	33 (2)	ns

BMI: body mass index; BP: blood pressure; CV: cardiovascular; GE: gastroenterological; HT: hypertension; ns: not significant. Continuous variables are expressed as mean ± standard deviation. Frequencies of categorical variables are expressed as percentages and the exact number of patients is indicated in brackets. Comparisons of paired groups were performed using Wilcoxon and McNemar tests with continuity correction for continuous variables and categorical variables, respectively. Significance was set at the 5% level.

**Table 4:** List of relevant features for the discriminant orthogonal partial least squares discriminant analysis (OPLS-DA) models of NT versus HT individuals (A) and of dippers versus non-dippers (B).

(A) NT vs. HT			(B) Dippers vs. Non-Dippers		
X-Variable in ppm	VIP	Feature ID	X-Variable in ppm	VIP	Feature ID
0.91	1.51	Butyrate	0.91	1.1	Butyrate
3.39	1.48	matrix	3.84	1.1	matrix
0.89	1.47	Butyrate	3.94	1.09	matrix
2.99	1.43	matrix	2.09	1.09	matrix
1.55	1.43	Butyrate	2.14	1.09	Propionate
2.14	1.39	Propionate	1.5	1.08	Butyrate
3.94	1.38	matrix	0.89	1.08	Butyrate
4.19	1.29	matrix	1.04	1.08	Propionate
0.54	1.25	matrix	2.04	1.08	matrix
0.64	1.24	matrix	1.06	1.08	Propionate
1.03	1.24	Propionate	1.93	1.06	Acetate
1.93	1.2	Acetate	1.74	1.06	matrix

ID: identity; HT: hypertensive; NT: normotensive; VIP: variable importance of projection.

## Data Availability

The metabolomics and metadata reported in this paper are available via MetaboLights (www.ebi.ac.uk/metabolights/MTBLS2553 accessed on 9 March 2021) study identifier MTBLS2553.

## Supplementary Materials

The following are available online at https://www.mdpi.com/article/10.3390/metabo11050282/s1, Table S1. Comparison of dietary habits between normotensive and hypertensive patients in 2020 male and female cohorts (p. 3); Table S2. Comparison of dietary habits between dippers and non-dippers in 2020 male and female cohorts (p. 4); Table S3: Comparison of blood pressure and dipping classification between the “2015 male cohort” and the “2020 male cohort” according to 24 h ABPM results based on the ESH criteria (p. 5); Table S4: Comparison of the main clinical characteristics between the “2020 male cohort” and the “2020 female cohort” (p. 6); Figure S1: Flowchart of the three cohorts (p. 7); Figure S2: Cumulated relative population abundance for the genera contributing to more than 1% of the GM composition for all patients from 2020 cohorts (A) Comparison between the GM profile of male patients in 2015 versus 2020 (B) (p. 8); Figure S3: Discriminant analysis (PCA) of stool metabolomes between the 2020 male cohort (male) and the 2020 female cohort (female) (p. 9); Figure S4: Trajectory of SCFAs according to BP status between 2015 and 2020 (p. 10).
