# Supplementary material for: Human Stool Metabolome Differs upon 24 h Blood Pressure Levels and Blood Pressure Dipping Status: A Prospective Longitudinal Study
Source: Metabolites. 2021 Apr 29;11(5):282. doi: 10.3390/metabo11050282 (PMC8146767; doi:10.3390/metabo11050282)

Human stool metabolome differs upon 24-hour blood pressure levels and blood pressure dipping status: a prospective longitudinal study

Supplementary Appendix

Justine Huart * (1,2), Arianna Cirillo * (3), Bernard Taminiau * (4,5), Julie Descy (6), Annie Saint-Remy (1), Georges Daube (4,5), Jean-Marie Krzesinski (1,2), Pierrette Melin (6), Pascal de Tullio § (3), François Jouret § (1,2)

Affiliations:

(1) Division of Nephrology, University of Liège Hospital (ULg CHU), University of Liège, Liège, Belgium

(2) Groupe Interdisciplinaire de Génoprotéomique Appliquée (GIGA), Cardiovascular Sciences, University of Liège, Liège, Belgium

(3) Center for Interdisciplinary Research on Medicines (CIRM), Metabolomics group, University of Liège, Liège, Belgium

(4) Fundamental and Applied Research Center for Animal & Health (FARAH), Veterinary Public Health. University of Liège, Liège, Belgium

(5) Laboratory for Food Microbiology. Department of Food Sciences, Faculty of Veterinary Medicine, University of Liège, Liège, Belgium

(6) Clinical Microbiology, University of Liège Hospital (ULg CHU), University of Liège, Liège, Belgium

* These authors contributed equally to this work

§ These authors share the senior authorship of this paper

Corresponding author: Justine Huart, Service de Néphrologie, CHU Sart Tilman, 4000 Liège, Belgium, Phone: ++3243667111, Fax: ++3243667205, justine.huart@chuliege.be

Table of contents

Table S1. Comparison of dietary habits between normotensive and hypertensive patients in 2020 male and female cohorts (p.3)

Table S2. Comparison of dietary habits between dippers and non-dippers in 2020 male and female cohorts (p.4)

Table S3: Comparison of blood pressure and dipping classification between the “2015 male cohort” and the “2020 male cohort” according to 24h ABPM results based on the ESH criteria (p.5)

Table S4: Comparison of the main clinical characteristics between the “2020 male cohort” and the “2020 female cohort” (p.6)

Figure S1: Flowchart of the 3 cohorts (p.7)

Figure S2: Cumulated relative population abundance for the genera contributing to more than 1% of the GM composition for all patients from 2020 cohorts (A). Comparison between the GM profile of male patients in 2015 *versus* 2020 (B) (p.8)

Figure S3: Discriminant analysis (PCA) of stool metabolomes between the 2020 male cohort (Male) and the 2020 female cohort (Female) (p.9)

Figure S4: Trajectory of SCFAs according to BP status between 2015 and 2020 (p.10)

Table S1. Comparison of dietary habits between normotensive and hypertensive patients in 2020 male and female cohorts

	**Normotension**	**Hypertension**	**P value**
**N**	19	7	
Brown rice, bread or pasta (instead of white) (%, N)	89.5 % (17)	100 % (7)	0.949
Yoghurt (%, N)	89.5 % (17)	85.7 % (6)	0.670
Vegetarian diet (%, N)	10.5 % (2)	14.3 % (1)	0.670
Fruits and vegetables (daily consumption) (%, N)	100 % (19)	100 % (7)	ns
Vegetable oil (instead of butter) (%, N)	84 % (16)	100 % (7)	0.670
Sugar (instead of sweetener) (%, N)	89.5 % (17)	71.4 % (5)	0.136
Salt (cooking) (%, N)	89.5 % (17)	100 % (7)	0.949

Ns: not significant. Frequencies of categorical variables are expressed as percentages and the exact number of patients is indicated in brackets. Yates Chi-square test was used. Significance was set at the 5% level**.**

Table S2. Comparison of dietary habits between dippers and non-dippers in 2020 male and female cohorts

2020 male and female cohorts	Dippers	Non-dippers	P value
**N**	11	13	
Brown rice, bread or pasta (instead of white) (%, N)	91 % (10)	92 % (12)	0.536
Yoghurt (%, N)	100 % (11)	77 % (10)	0.278
Vegetarian diet (%, N)	18.2 % (2)	7.7 % (1)	0.876
Fruits and vegetables (daily consumption) (%, N)	100 % (11)	100 % (13)	ns
Vegetable oil (instead of butter) (%, N)	82 % (9)	92 % (12)	0.876
Sugar (instead of sweetener) (%, N)	80 % (8)	92 % (12)	0.346
Salt (cooking) (%, N)	82 % (9)	100 % (13)	0.387

Ns: not significant. Frequencies of categorical variables are expressed as percentages and the exact number of patients is indicated in brackets. Yates Chi-square test was used. Significance was set at the 5% level**.**

Table S3: Comparison of blood pressure and dipping classification between the "2015 male cohort" and the "2020 male cohort" according to 24h-ABPM results based on the ESH criteria

**2015 male cohort**				**2020 male cohort**		
**No change NT-NT**						
	**24h-BP (S/D/M)**	**Day BP (S/D/M)**	**Night BP (S/D/M)**	**24h-BP (S/D/M)**	**Day BP (S/D/M)**	**Night BP (S/D/M)**
#2	116/74/87	124/81/94	106/64/76	102/66/77	104/68/80	99/64/75
#3	112/66/82	119/72/88	97/54/70	125/75/92	128/78/95	119/66/84
#4	123/79/92	130/84/98	110/67/81	116/71/85	122/78/91	104/59/73
#5	114/76/89	118/80/92	108/71/83	106/74/84	107/76/86	105/71/82
#9	127/75/93	130/79/97	122/66/84	120/71/90	122/72/91	117/70/89
#11	118/70/85	124/76/91	109/60/76	111/67/82	118/72/88	98/56/71
#12	125/75/91	130/80/95	113/63/80	123/79/94	127/83/99	113/70/84
#14	106/64/79	111/68/84	96/55/69	104/63/77	106/66/81	97/55/70
**No change HT-HT**						
#6	129/79/94	140/88/104	110/64/77	122/77/92	126/81/97	114/67/82
#7	120/80/93	124/84/97	104/66/78	130/84/99	135/89/104	109/67/80
**Change HT-NT**						
# 8	128/77/94	135/83/100	112/64/80	125/76/92	128/79/95	111/65/82
#15	127/76/91	137/86/101	110/61/76	120/72/87	125/77/92	105/58/73
**Change NT-HT**						
#1	124/73/89	131/80/95	111/61/78	128/86/105	128/87/105	125/82/102
#10	125/78/93	126/80/94	118/75/88	129/81/97	134/85/101	116/70/86
#13	121/78/92	122/79/93	121/77/91	122/80/93	124/82/95	113/72/85
#16	127/78/93	129/82/96	121/68/85	132/80/104	136/83/107	117/69/91
**2015 male cohort**				**2020 male cohort**		
**No change Dip-Dip**						
		**Ratio ND**			**Ratio ND**	
# 4		0.85			0.85	
# 7		0.84			0.81	
# 8		0.83			0.87	
# 11		0.88			0.83	
# 12		0.87			0.89	
# 15		0.80			0.84	
**No change NonDip-NonDip**						
# 5		0.92			0.98	
# 9		0.94			0.96	
# 13		0.99			0.91	
**Change NonDip-Dip**						
# 10		0.94			0.87	
# 16		0.94			0.86	
**Change Dip-NonDip**						
# 1		0.85			0.98	
# 2		0.85			0.95	
# 3		0.82			0.93	
# 14		0.86			0.92	

BP: blood pressure; D: diastolic BP; Dip: dippers; HT: hypertensive; M: mean BP; NonDip: non-dippers; NT: normotensive; Ratio ND: ratio night-day systolic BP; S: systolic BP

Table S4: Comparison of the main clinical characteristics between the "2020 male cohort" and the "2020 female cohort"

	2020 male cohort	2020 female cohort	P value
**N**	16	10	
Age (years)	52.3 ± 12.4	46.5 ± 13.2	0.246
BMI (kg/m²)	24.6 ± 2.6	22.3 ± 2.2	0.037
Smokers (%, N)	6.2 (1)	10 (1)	0.683
Alcohol (glass/week)	6.4 ± 7	1.2 ± 1.6	0.021
Family HT (%, N)	56.2 (9)	30 (3)	0.367
Diabetes (%, N)	6.2 (1)	0	0.808
CV history (%, N)	18.7 (3)	20 (2)	0.665
GE history (%, N)	31.2 (5)	30 (3)	0.711
24h Systolic BP (mmHg)	120 ± 9	113 ± 8	0.065
24h Diastolic BP (mmHg)	75 ± 7	69 ± 7	0.048
24h Mean BP (mmHg)	91 ± 8	85 ± 7	0.170
Anti-HT treatment (%, N)	6.2 (1)	10 (1)	0.683
Non-dippers (%, N)	50 (8)	30 (3)	0.315

BMI: body mass index; BP: blood pressure; CV: cardiovascular; GE: gastroenterological; HT: hypertension; ns: not significant. Continuous variables are expressed as mean ± standard deviation. Frequencies of categorical variables are expressed as percentages and the exact number of patients is indicated in brackets. Mann-Whitney U test and Yates Chi-square test were used to compare continuous variables and categorical variables, respectively. Significance was set at the 5% level.

Figure S1: Flowchart of the 3 cohorts

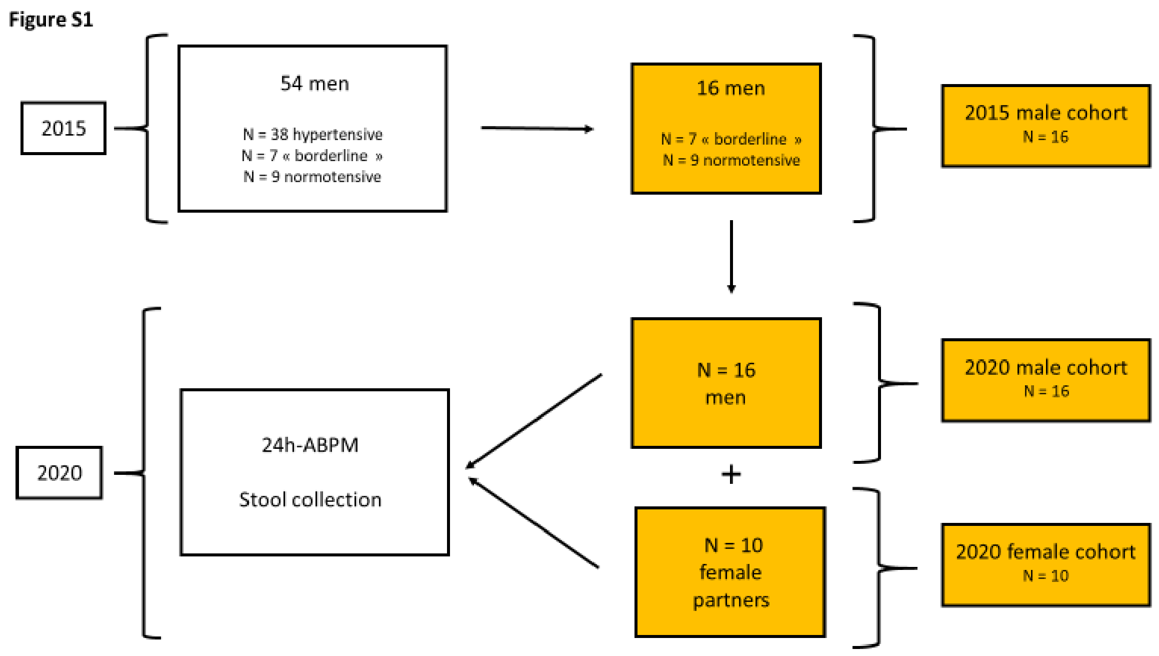

24h-ABPM; 24h-ambulatory blood pressure monitoring

Figure S2: Cumulated relative population abundance for the genera contributing to more than 1% of the GM composition for all patients from 2020 cohorts (A). Comparison between the GM profile of male patients in 2015 *versus* 2020 (B)

A.

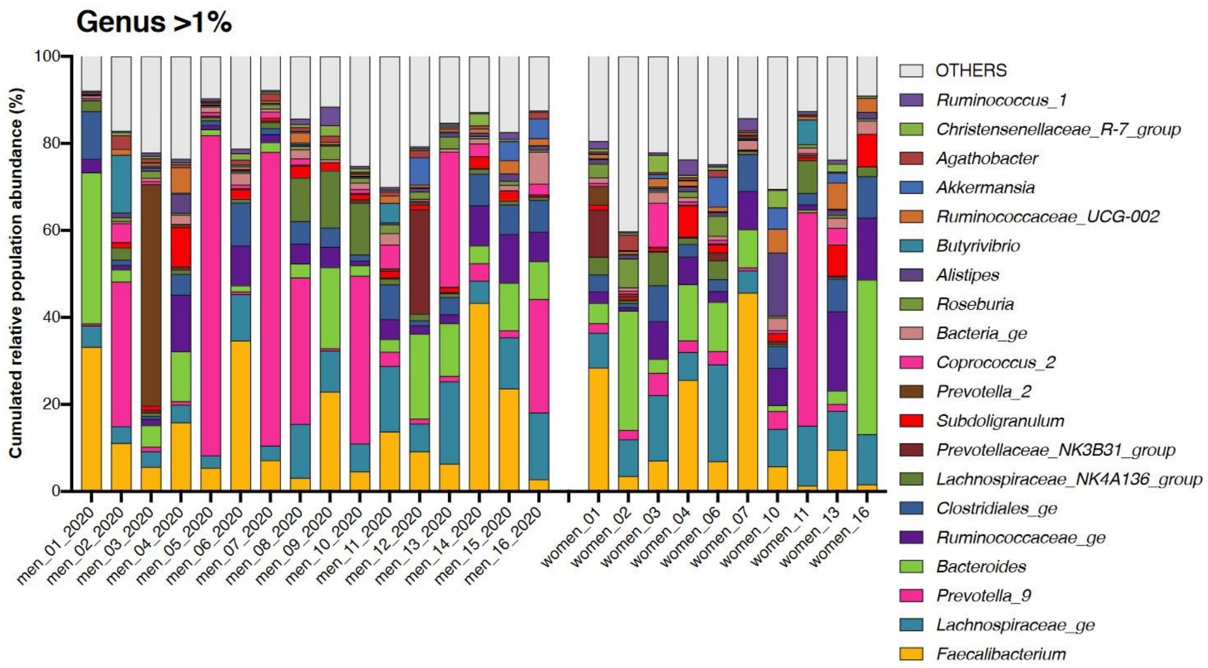

B.

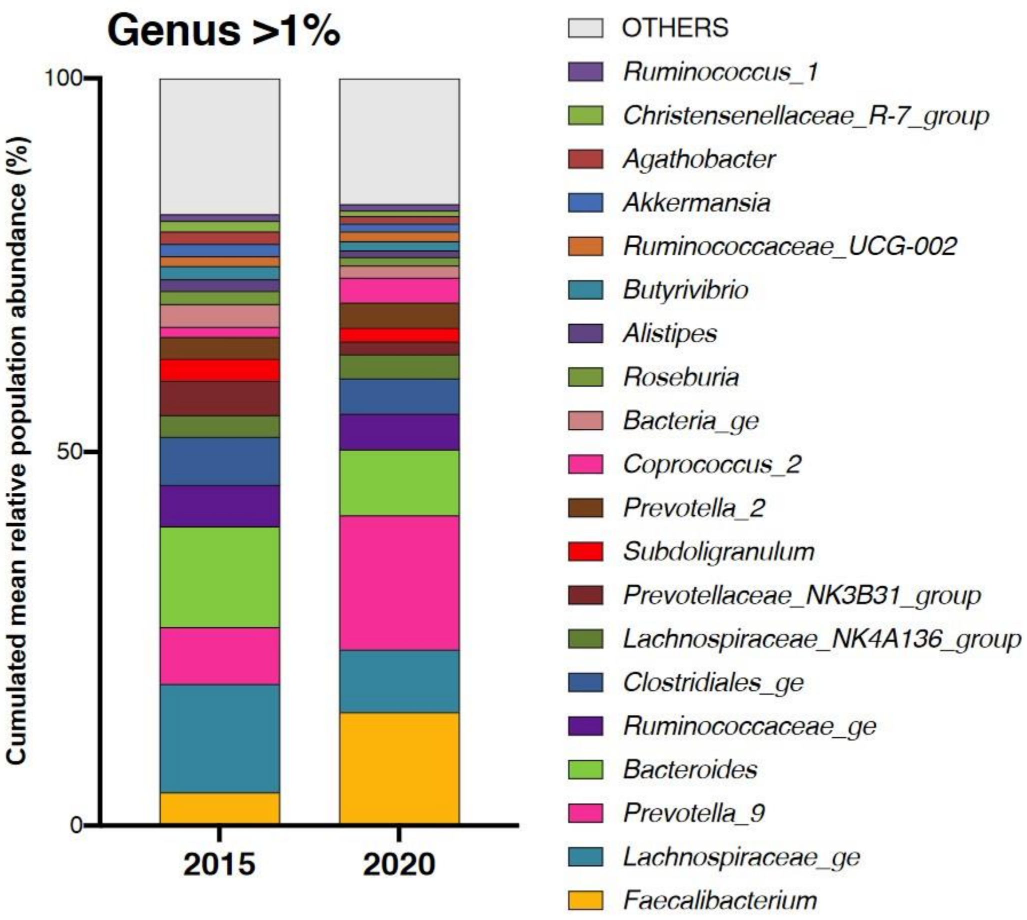

Figure S3: Discriminant analysis (PCA) of stool metabolomes between the 2020 male cohort (Male) and the 2020 female cohort (Female).

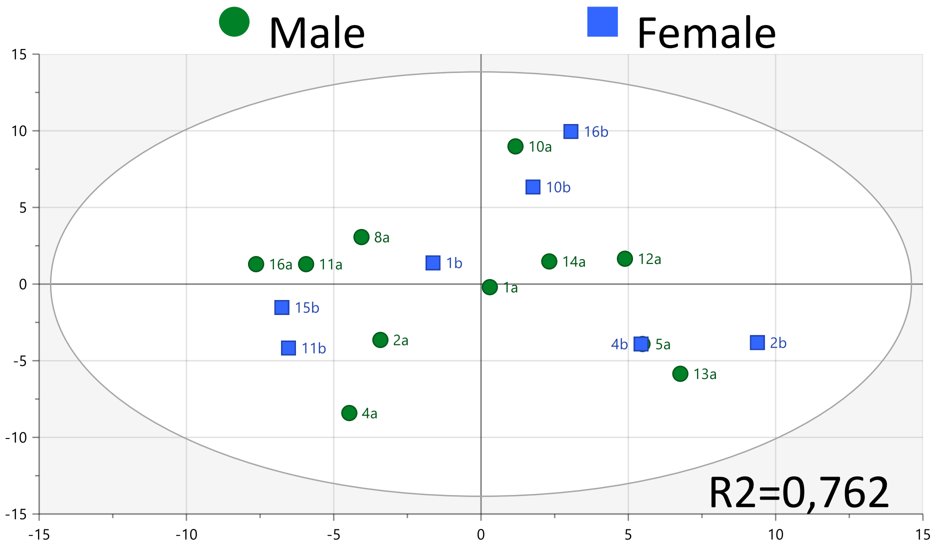

Figure S4: **Trajectory of SCFAs according to BP status between 2015 and 2020**

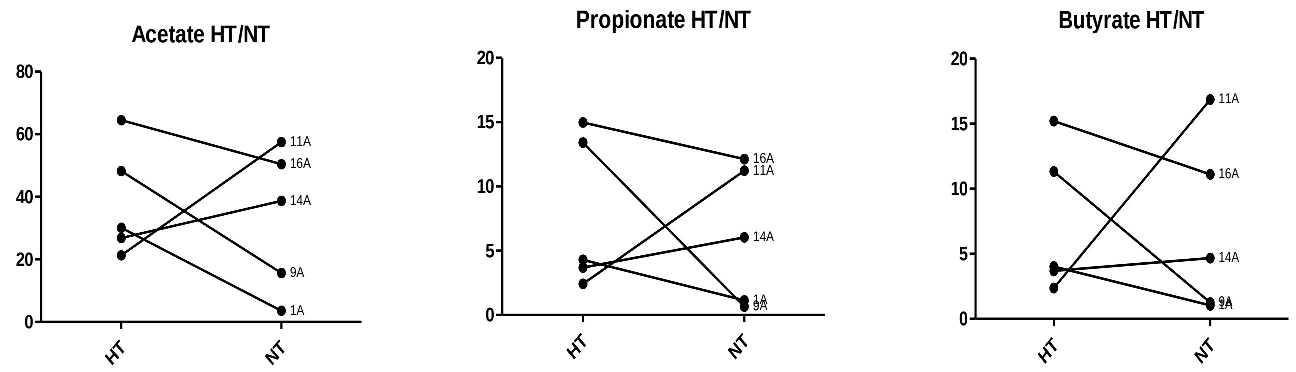

Supplement: Supplementary file 1 [file metabolites-11-00282-s001.zip › Huart_supplement_Metabolites-R1.pdf]
